# Clinical effects of early debridement, internal fixation, and Masquelet technique for childhood chronic haematogenous osteomyelitis of long bones

**DOI:** 10.1186/s13018-022-03478-7

**Published:** 2023-01-05

**Authors:** Jian Shi, Xiaoyong Yang, Muguo Song, Xijiao Zhang, Yongqing Xu

**Affiliations:** Department of Orthopaedics, 920 Hospital of the Joint Logistics Support Force of the PLA, 212 Daguan Road, Kunming, 650032 China

**Keywords:** Chronic osteomyelitis, Paediatric, Internal fixation, Masquelet technique

## Abstract

**Background:**

Childhood chronic haematogenous osteomyelitis (CCHOM) is a severe condition in paediatric patients. The optimal timing of debridement and the subsequent method of bone reconstruction in CCHOM patients remain controversial. The purpose of this study was to assess the treatment efficacy of Masquelet technique with early debridement and internal fixation in CCHOM of long bones.

**Methods:**

Between January 2016 and January 2021, a total of 21 patients (18 males, 3 females) with CCHOM of long bone were included. The mean age was 10.4 years (range, 2–18 years). All cases were treated by a two-stage surgical protocol of Masquelet technique. In the first stage, aggressive debridement, sequestrectomy, and inducing membrane by bone cement spacer were performed after definite diagnosis. In the second stage, cement spacer was removed, and autologous and allogeneic bone was grafted. Internal fixation was used for the first and/or second stage depending on stability requirements. The patients’ clinical and imaging results were retrospectively analysed.

**Results:**

The mean follow-up was 31.7 months (range, 21–61 months). None of the patients experienced recurrence of infection. Radiographic bone union time was 4.3 months (range, 2.5–11 months). Five cases underwent re-operation due to complications such as bone resorption or refracture. By the last follow-up visit, bones had healed and all of the patients had resumed daily living and sports activities.

**Conclusion:**

The Masquelet technique with early debridement and internal fixation is a viable surgical method for the management of large long bone defects of CCHOM patients.

## Background

Although childhood chronic haematogenous osteomyelitis (CCHOM) has become less prevalent in developed nations, it remains a major source of morbidity and a ponderous burden on public health care in developing countries [1]. In southwest China, haematogenous osteomyelitis accounts for 17.9% of all cases of chronic osteomyelitis [2]. CCHOM is frequently accompanied by clinical manifestations such as pain, chronic sinus tracts, bone exposure, and pathological fractures. Physical manifestations of the disease can lead to secondary problems for these paediatric patients, such as psychological and economic problems [3].

CCHOM mainly involves metaphyses of long bones in an early stage and occurs at a single site in majority of cases [3]. It may lead to extensive necrosis of bone and formation of sequestra. Surgery is the most important means of treating CCHOM. However, there are many controversies regarding the optimal surgical method. Sequestrectomy, external fixator, bone graft or Ilizarov technique, and systemic antibiotic treatment are standard treatment methods [4–13]. Although many patients are cured, these treatment methods are often accompanied by complaints such as poor patient tolerance, high complication rates, and long-term recurrence rates (Table 1). The Masquelet technique, also known as the induced membrane technique, is a two-stage approach used in the reconstruction of bone defects. It involves filling the defect with polymethyl methacrylate (PMMA) cement spacer to induce a membrane in the first stage, and removing the spacer and grafting cancellous bone in the second stage [14]. It has widely been used in the treatment of chronic osteomyelitis [15, 16]. The technique has many advantages such as a high rate of infection eradiation, a relatively short time of bone union, and few complications, even for critical size bone defects [17]. Combined with internal fixation, it can provide better comfort for the patients and does not increase the risk of infection recurrence [18]. The clinical effect has been reported in adults [18]. However, in children, the outcomes of this technique are still uncertain. The aim of this study was to assess the efficacy of the Masquelet technique with early debridement and internal fixation for the treatment of CCHOM of long bones.

**Table 1:** Literature review of childhood chronic haematogenous osteomyelitis (CCHOM) of long bones

Work	Cases (*n*)	Mean age (years)	Gender (M/F)	Location (*n* or %)	Surgical methods used	Follow-up (mean)	Results	Complications at the last follow-up
Daoud et al. [4]	34	7.7	17/17	Tibia: 24 Femur: 8 Humerus: 2	Sequestrectomy, Debridement, Immobilisation Corticocancellous iliac grafts	37 months		Limb length discrepancy, Fracture, Axial deformity, Non-union
Liu et al. [5]	11	14	7/4	Humerus: 11	Osteotomy, Callus distraction by monolateral external fixator	106 months	Excellent: 7 Good: 3 Poor:1	Pin track infection, Local inflammation, Flexion contracture of elbow, Pin loosening, Inferior subluxation of the glenohumeral joint
Hoang et al. [6]	6	9.8	6/0	Tibia: 6	Debridement, Free gracilis muscle flap,	3 years	Satisfactory: 6	No complication
Kucukkaya et al. [7]	7	7.2	5/2	Tibia: 7	Sequestrectomy, Debridement, Ilizarov technique	4.6 years	Excellent: 7	No complication
Stevenson et al. [8]	145	10		Tibia: 46% Femur: 26% Humerus: 10%	Drilling and curettage of abscess, Sequestrectomy, Stabilisation, Reconstruction of bone defect	At least 3 years		
Beckles et al. [9]	167	8	102/65	Tibia: 79 Femur: 47 Humerus: 18	Sequestrectomy, Drilling Curettage, Incision and drainage of abscess, Bone grafting, Bone transport	At least one year		Infection recurrence, Below-knee amputation
Ellur et al. [10]	34	4	22/9	Tibia: 17 Femur: 12 Humerus: 1 Metacarpal: 1	Debridement, Antibiotic-impregnated calcium sulphate beads	42 months	No infection recurrence No reoperation No systemic adverse reaction	Length discrepancy (< 1 cm)
El-Rosasy et al. [11]	14	9.2 years	9/5	Tibia: 14	Ilizarov techniques, Fibular osteotomy	36.9 months	Satisfactory 12 Unsatisfactory 2	Refracture, Limb shortening
Wirbel et al. [12]	16 (out of 11 patients)				Debridement, Sequestrectomy External fixator Vacuum-assisted closure (VAC) system, Coverage of the soft tissue, Fibula or rib transplantation	24 months		Functional restrictions, Length discrepancy
Lauschke et al. [13]	30 (out of 24 patients)			Tibia: 30	Drainage of abscess, Cortical fenestration, Sequestrectomy, A composite transposition of the ipsilateral fibula	2 years		Restriction of motion, Limb shortening
Wang et al. [27]				Tibia	Debridement, Masquelet technique	25 months	Bone union, No infection recurrence	Small bone diameter, Limitation of knee activity

## Methods

**Fig. 1:**
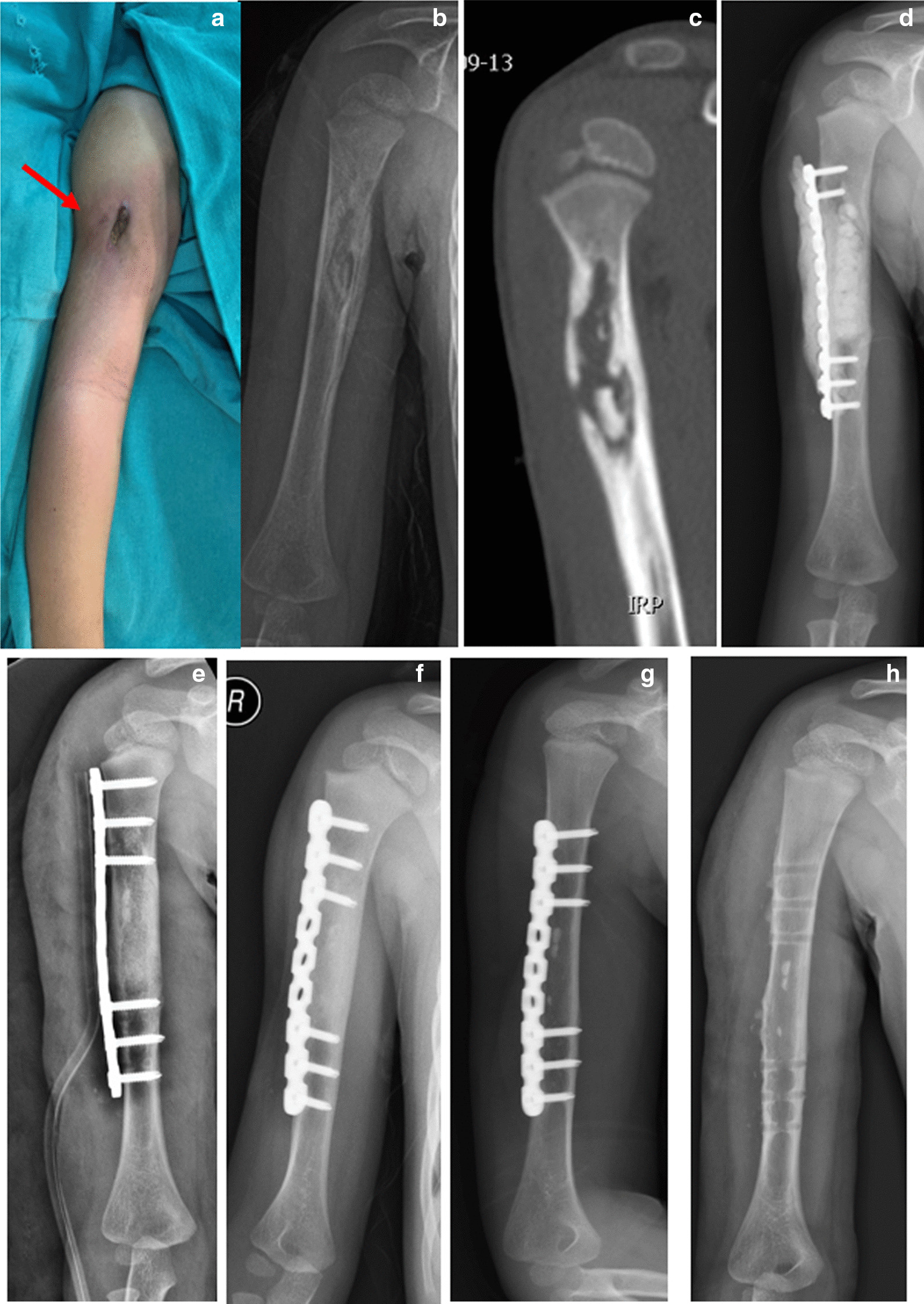
Case 5: a 3-year-old boy who has recently presented with recurrent swelling and drainage from his right upper arm. At the age of 2, he underwent surgery due to swelling, redness, and pain with no preceding trauma. Afterward, a sinus tract formed at the incision (red arrow). **a** Pre-operative presentation of an affected limb. **b** Anteroposterior (AP) radiograph shows chronic osteomyelitis of the right humerus. **c** CT shows a sequestrum. **d** In the first stage of surgery, debridement and locking plate were performed and antibiotic-loaded bone cement was used to fill the bone defect area. **e** AP radiograph after bone graft in the second stage of surgery. **f** AP radiograph shows bone defect healed at 3 months after bone grafting. **g** AP radiograph shows bone defect remodelled at 26 months after bone grafting. **h** AP radiograph shows that the internal fixation was removed at 26 months after bone grafting

**Fig. 2:**
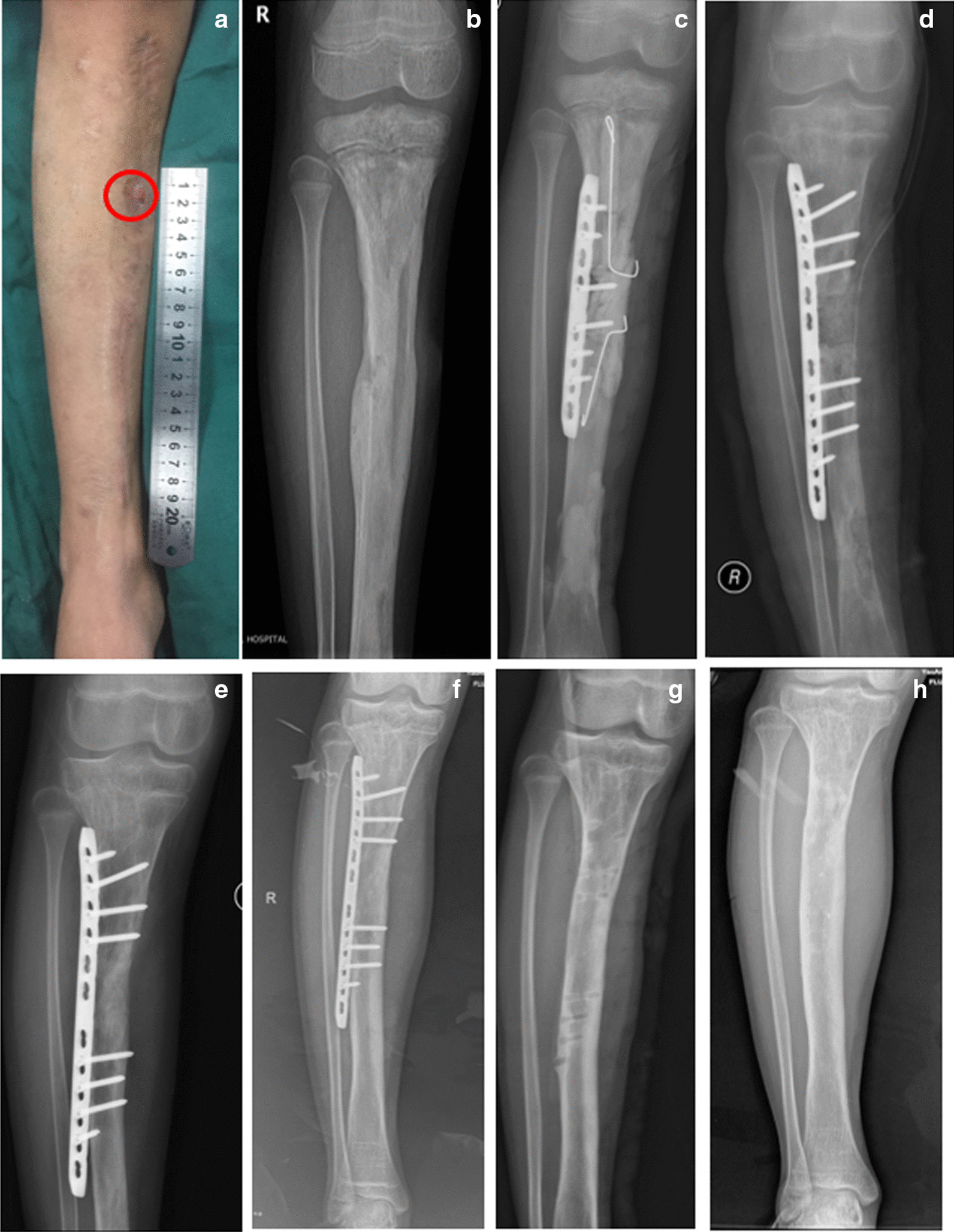
Case 19: a 10-year-old boy with recurrent pain and drainage from his right tibia. Three months before, he underwent surgery twice due to swelling, redness, and pain with no preceding trauma. However, the drainage from sinus tract (red circle) and pain did not disappear after the operations. **a** Pre-operative presentation of the right lower leg. **b** Anteroposterior (AP) radiograph shows chronic osteomyelitis of the right tibia. **c** In the first-stage surgery, debridement and internal fixation were performed, and antibiotic-loaded bone cement was used to fill the bone defect and medullary cavity. **d** AP radiograph after bone graft in the second stage of surgery. **e** AP radiograph shows bone defect healed at 3 months after bone grafting. **f** AP radiograph shows bone defect remodelled at 23 months after bone grafting. **g** AP radiograph shows that internal fixation was removed at 26 months after bone grafting. **h** AP radiograph shows the right tibia at 47 months after bone grafting

After the approval from the institutional review board, the procedures were performed from January 2016 to January 2021 at the Department of Orthopaedic Surgery, 920 Hospital of Joint Logistic Support Force of PLA, China. The inclusion criteria were as follows: (1) chronic osteomyelitis of long bones, which was confirmed by clinical features and imaging (plain radiographs, CT, and MRI); (2) lack of history of fracture or surgery before the onset of bone infection at the affected site; (3) type III or IV Cierny–Mader anatomic type; (4) treatment with the Masquelet technique; (5) bone defect was stabilised by internal fixation in the first or the second stages; (6) age less than 18 years; (7) A or B-host Cierny–Mader physiologic class. Patients with insufficient follow-up information were excluded. Table 2 summarises the patients’ data (Figs. 1 and 2).

**Table 2:** Patients’ demographic and clinical data at the last follow-up

Patient number	Sex	Age (years)	Location	Cierny–Mader type	Fixation		Follow-up (months)	Radiological bone union (months)	Full weight-bearing (months)	Outcomes
					1st	2nd				
1	F	13	Tibia	III	None	IP	25	3	3	Good
2	M	17	Tibia	IV	IP	IP + Nail	36	7	8	Bone resorption (local)
3	M	3	Ulna	IV	IP	IP	37	11	12	Bone resorption (segmental)
4	M	18	Radius	IV	IP	IP	26	4	5	Good
5	M	3	Humerus	III	IP	IP	46	3	4	Good
6	M	14	Femur	III	IP	IP	33	4	6	Good
7	F	8	Femur	III	IP	IP	27	3	4	Good
8	M	6	Femur	III	IP	IP	24	3	4	Good
9	M	15	Tibia	IV	IP	Nail	36	6	9	Good
10	M	13	Femur	III	IP	IP	22	5	6	Good
11	M	2	Tibia	III	None	IP	22	2	3	Good
12	M	11	Tibia	IV	IP	IP	25	3	4	Good
13	M	12	Tibia	IV	IP	IP	21	4	5	Refracture
14	F	10	Tibia	III	IP	IP	29	2.5	3	Good
15	F	7	Femur	III	None	IP	27	3	3	Good
16	M	9	Tibia	IV	IP	IP	24	3	4	Good
17	M	11	Tibia	IV	IP	IP	44	9	12	Broken plate
18	M	9	Tibia	III	None	IP	44	3	6	Varus deformity of the ankle joint
19	M	10	Tibia	IV	IP	IP	61	3	4	Good
20	M	16	Fibula	IV	IP	IP	30	3	4	Good
21	M	11	Tibia	IV	IP	IP	26	5	5	Good

*F* female; *M* male, *IP* internal plate, *nail* intramedullary nail

### Surgical technique

All of the patients were treated with the Masquelet technique by a two-stage procedure.

The first stage began with aggressive debridement of all infected, necrotic tissues. Sinus was resected; sequestrum was removed; and medullary cavity was rimmed. The extent of debridement was determined by pre-operative radiographs, CT, and bone scintigraphy. Debridement was carried out until punctate bleeding (Paprika sign) was seen at the bony and soft tissue margins. After abundant lavage, a narrow locking plate was used for the stabilisation of the bone defect. In principle, when the bone defect was segmental or for the local bone defect whose width exceeded one-third of the circumference of the bone cortex, internal fixation was used. Fluoroscopy was used to confirm that the screw was not placed in the epiphyseal plate and that the alignment and length of the affected bone were appropriate. Then, we placed antibiotic-loaded (Vancomycin, Lilly, 2–4 g per 40 g cement) PMMA bone cement (PALACOS^®^R+G, HERAEUS, Germany) into the bone defect and then coated the plate and both bone ends of the defect with the cement. Two drainage tubes were used for 7–14 days. The deep tissue was harvested for bacterial culture and pathological examination. Weight bearing was forbidden on the affected limb.

All of the patients received intravenous broad-spectrum antibiotics for two weeks post-operatively. According to the results of bacterial culture, they were replaced with sensitive antibiotics, which were then changed to oral antibiotics for 4 weeks. White blood cell (WBC) count, erythrocyte sedimentation rate (ESR), and C-reactive protein (CRP) level were examined every two weeks. The next stage of surgery was performed after 6–8 weeks. The prerequisite was that WBC count, ESR, and CRP level were normal for 2–3 times. During the second stage of procedure, the membrane was incised sharply longitudinally to reach the bone cement, and the antibiotic-loaded bone cement and the locking plate were removed carefully, avoiding damage to the induced membrane. After rinsing thoroughly, a new similar locking plate was used for defect fixation with simultaneous osteosynthesis performed as the first stage. The bone was decorticated proximally and distally until bone bleeding was obtained. Then, the bone defect was filled with autogenous cancellous bone within the induced membrane. The graft was morselised into very small grafts measuring 1–2 mm^3^. If autologous cancellous bone from the iliac crests was insufficient, allogeneic bone (BIO-GENE, Beijing Cojoing, China) was added. The allogeneic bone was placed in the middle of the defect, and the autologous bone was placed at the surrounding area near the induction membrane. Before using, the allograft was soaked in the blood harvested from the donor site for at least 30 min.

Sensitive antibiotics were continued for 2 weeks after the second stage, and weight bearing of the affected limb was restricted unless allowed by the surgeons.

### Follow-up

All of the patients had regular clinical and radiological follow-up every month after surgery until the bone healing. Thereafter, the patients were followed up every three months. Radiological bone union was defined as at least three continuous cortices visible on anteroposterior (AP) and lateral radiographs of the bone defect. The follow-up radiographs were independently assessed by two authors in an unblinded fashion. Disagreements between the two authors were judged by another author.

During each follow-up visit, the patients underwent a clinical evaluation and laboratory analyses. WBC, CRP, and ESR, as well as other clinical features (such as discharge, redness and swelling, warmth, and pain) were assessed to exclude the recurrence of infection. Furthermore, range of motion (ROM) of joint in the affected limb and growth disturbances were recorded at the final follow-up to determine the treatment effect. Complications were assessed by the surgeons involved in the treatment of the patients. At the last follow-up, if the patient had no symptoms of recurrent infections such as local redness, swelling, pain, and sinus in the affected limb, the radiologic imaging showed good bone healing without deformity, and there was no other complication; the treatment outcome could be considered “good”.

## Results

The study included a total of 21 consecutive children (18 males, 3 females) with CCHOM of long bones. The mean patients’ age at the time of the first hospitalisation was 10.4 (range, 2–18) years. The femur was involved in five cases, the tibia in 12 cases, the humerus in one case, the radius in one case, the fibula in one case, and the ulna in one case. Cierny–Mader Type III and Type IV were observed in 10 cases and 11 cases, respectively. All patients had A-host Cierny–Mader physiologic class. Thirteen cases had sinus tracts, and 12 cases had a history of surgery in other hospitals.

Two patients required repeated operation due to recurrent infection during the interval between the first and the second stage, and infection was controlled after re-debridement. Nine patients had positive bacterial cultures. The causative pathogen was *Staphylococcus aureus* in six cases, *Enterobacter cloacae* in two cases, and *Staphylococcus epidermidis* in one case.

The volume of bone defect was estimated based on CT measurement before the second-stage surgery. The average volume of bone defect was 36.4 cm^3^ (9–78 cm^3^). Four patients were implanted with autologous bone, and 17 patients were implanted with autologous plus allograft in different proportions (16–50%), of which five patients were implanted with allograft more than 25% of the total volume. The mean follow-up period after the second stage of surgery was 31.7 months (range, 21–61 months). Table 2 summarises the management procedures and results. The mean radiographic bone union occurred in 4.3 months (range, 2–11 months), and full weight bearing was noted at 5.5 months (range, 3–12 months).

Two cases underwent bone re-grafting due to partial or segmental bone resorption at the defect. The proportion of allograft bone was 38% and 33% in these two cases. In one case, internal fixation was broken due to an accidental fall, and surgery was required to replace it with a new plate, which resulted in bone union delay of 9 months. One case with distal tibia osteomyelitis had a varus deformity (about 17°) of the ankle joint at 16 months after grafting, but did not have pain, lameness, or other symptoms. In one case, a refracture occurred at the bone defect due to a fall one month after removal of the internal fixation. Reduction, fixation, and grafting were performed again, and the fracture healed six months after surgery.

By the last follow-up visit, all of the patients achieved bone union and were pain-free; their inflammatory markers remained within the normal range; no infection recurrence was noted; and they resumed daily living and sports activities.

## Discussion

We showed that the early debridement and internal fixation with the Masquelet technique could effectively control infection, relieve patients’ symptoms, achieve bone healing, and facilitate early functional exercise in patients with CCHOM.

The Beit-CURE (BC) classification is the first classification specific for CCHOM [1, 8–10]. It considers the presence of both sequestrum and involucrum. Some studies have confirmed that the BC classification can guide surgical strategy and help predict length of inpatient treatment and number and type of procedures required [8–10]. However, in our hospital, most of the patients had undergone one or more surgeries in local hospitals before they were admitted. According to the BC classification, they should be categorised as unclassifiable, and therapy recommendations are not conceivable. Cierny–Mader’s classification is based on the extent of infection (medullary, superficial, localised, diffuse) and host status (healthy, compromised immune system, failed immune system) [19, 20]. This is the most widely used classification for chronic osteomyelitis. It represents the pathological progression of osteomyelitis and is useful in planning treatment strategy [13]. Although it is mainly suitable for adult patients, it can be applied to the paediatric population as well; namely, in this study, we adopted different strategies of debridement and stabilisation according to this classification.

The timing of debridement of CCHOM is controversial. Some authors believe that the intact involucrum develops, which can provide good stability, before sequestrectomy is required to reduce the risk of complication such as pathological fractures, deformities, and segmental bone defects [5, 11, 13]. Other authors, however, advocate for the early debridement to control infection, create a better environment for the periosteum to respond, and minimise damage to the surrounding soft tissues [1, 5]. In our cases, due to the application of internal fixation, the stability of the affected limb could be maintained; thus, debridement could be performed after diagnosis is confirmed, regardless of whether involucrum has formed or not. This undoubtedly shortened the treatment period and accelerated the recovery of the children.

The Masquelet’s technique is regarded as the “gold standard” for treating various types of long bone defects in adults and children [21, 22]. Auregan et al. [23] found that paediatric patients treated with the Masquelet technique had a 58% success rate, which increased to 87% when iterative surgery was considered. Canavese et al. [24] and Rousset et al. [25] reported five and eight children with chronic osteomyelitis treated by the Masquelet technique, respectively, and achieved satisfactory treatment results. Shen et al. treated 26 children with chronic osteomyelitis with the Masquelet technique, and the bone defects were healed in 4.0–5.0 months after the operation. Wang et al. [26] achieved good clinical efficacy in treating chronic osteomyelitis in both adult and paediatric patients. These reports confirm that the Masquelet technique is an effective treatment for chronic osteomyelitis in children, but it is mainly used for post-traumatic osteomyelitis, while there are no specific studies on CCHOM.

The application of internal fixation is typically contraindicated in the treatment of chronic osteomyelitis. However, some scholars have used antibiotic-loaded bone cement–coated plate as a temporary fixation after debridement, which can kill planktonic bacteria and inhibit the formation of biofilms [16, 18]. In the second stage, the application of internal fixation for bone reconstruction can reduce the burden of care, avoid pin tract infections, and allow early functional exercise. In this study, for the Cierny–Mader type III patients, according to the protocol by Kinik et al. [27], more than 30% of cortical bone removal for debridement necessitates prophylactic fixation to prevent iatrogenic fracture risk. For the Cierny–Mader type IV, we used locking plates for internal fixation in the first and second stages. This did not increase the chance of infection recurrence, but it reduced the difficulty of postoperative care and improved the comfort of the child. In the second stage, significant solidification of the bone graft was seen 2–3 months after the second stage of surgery, so that full weight bearing could be gradually achieved. Children could return to society early and participate in classroom learning and activities, which is beneficial to the physical and mental development, especially in younger patients.

In the second stage of the Masquelet's technique, the need for a large amount of autologous cancellous bone graft to reconstruct bone defects is a limiting factor, especially in very young children. The application of some alternative materials has been tried in clinical practice. Fitoussi et al. [28] used autologous cancellous bone particles combined with autologous fibula scaffold to fill the bone defect in the second stage for eight children with post-operative bone defect larger than 15 cm due to primary malignant tumour. All of the bone defects healed within 5.6 (range, 4–8) months. Gouron et al. [29] performed bone reconstruction in 14 children with trauma, tumour resection, or tibial congenital pseudarthrosis. They added allograft bone, biphasic calcium phosphate (BCP), or tibial bone strut to increase the graft volume. Bone union was achieved in 9.5 (range, 2–25) months. Canavese et al. [24] and Rousset et al. [25] used β-tricalcium phosphate (BTP) as a bone graft substitute. Their results showed that BTP was even more effective in osteogenesis than bone graft. Shen et al. [30] used bone marrow concentrator–modified allograft or bone marrow aspirate–mixed allograft to improve the osteogenic ability of allograft. In this study, allograft bone was added as an alternative graft material to increase the graft volume. Prior to use, in accordance with the method reported by Gouron [22, 28], the allograft was immersed in blood from the donor site. Two patients had resorption, and we believe the higher proportion of allogeneic bone was the main reason. Therefore, it is recommended that the proportion of allograft bone does not exceed one-third to avoid hindering consolidation according to an empirical advice of Masquelet [21].

There were several limitations to our study: (1) small sample size; (2) different skeletal sites involved; (3) no comparison with other treatment methods. Thus, additional studies are mandatory.

## Conclusion

CCHOM is a relatively complex disease, and once the diagnosis is confirmed, early debridement can effectively prevent further bacterial damage to the bone. Depending on the infection, the choice of an appropriate surgical method with aggressive debridement, the use of antibiotic-loaded bone cement to induce membrane formation, and then reconstruction by bone grafting in the second stage can achieve satisfactory results in patients with CCHOM. Early debridement can shorten the course of treatment; internal fixation can provide a stable osteogenic environment; and induced membrane in the Masquelet technique can rapidly promote the graft corticalisation. These measures are conducive to early joint functional exercise and reduce joint functional damage.

## Data Availability

The data presented in this study are available in the article or supplementary material.

## Abbreviations

CCHOM: Childhood chronic haematogenous osteomyelitis
WBC: White blood cell
CRP: C-reactive protein
ESR: Erythrocyte sedimentation rate
PMMA: Polymethyl methacrylate
ROM: Range of motion
BCP: Biphasic calcium phosphate
BTP: β-tricalcium phosphate
